# The Effect of Random Roughness on the Electromagnetic Flow in a Micropipe

**DOI:** 10.3390/mi14112054

**Published:** 2023-11-02

**Authors:** Zhili Wang, Yanjun Sun, Yongjun Jian

**Affiliations:** 1 School of Mathematical Science, Inner Mongolia University, Hohhot 010021, China or z.l.wang@139.com (Z.W.); sunyanjun@imufe.edu.cn (Y.S.); 2 School of Statistics and Mathematics, Inner Mongolia University of Finance and Economics, Hohhot 010070, China

**Keywords:** random roughness, electromagnetic flow, roughness function, micropipe

## Abstract

The features of stationary random processes and the small parameter expansion approach are used in this work to examine the impact of random roughness on the electromagnetic flow in cylindrical micropipes. Utilizing the perturbation method, the analytical solution until second order velocity is achieved. The analytical expression of the roughness function *ζ*, which is defined as the deviation of the flow rate ratio with roughness to the case having no roughness in a smooth micropipe, is obtained by integrating the spectral density. The roughness function can be taken as the functions of the Hartmann number *Ha* and the dimensionless wave number *λ*. Two special corrugated walls of micropipes, i.e., sinusoidal and triangular corrugations, are analyzed in this work. The results reveal that the magnitude of the roughness function rises as the wave number increases for the same Ha. The magnitude of the roughness function decreases as the *Ha* increases for a prescribed wave number. In the case of sinusoidal corrugation, as the wave number *λ* increases, the Hartmann number *Ha* decreases, and the value of *ζ* increases. We consider the *λ* ranging from 0 to 15 and the *Ha* ranging from 0 to 5, with *ζ* ranging from −2.5 to 27.5. When the *λ* reaches 15, and the *Ha* is 0, *ζ* reaches the maximum value of 27.5. At this point, the impact of the roughness on the flow rate reaches its maximum. Similarly, in the case of triangular corrugation, when the *λ* reaches 15 and the *Ha* is 0, *ζ* reaches the maximum value of 18.7. In addition, the sinusoidal corrugation has a stronger influence on the flow rate under the same values of *Ha* and *λ* compared with triangular corrugation.

## 1. Introduction

Many microfluidic devices are used in the chemical, pharmaceutical, biological, and other industries to separate and transfer substances. Electromagnetic fluid micropumps [1,2] and electroosmotic micropumps [3,4,5,6] are the two primary types of microfluidic devices. Due to its vast potential for use in the field of separation and analysis, magnetic fluid control technology is an important research direction [7,8,9]. Jang and Lee [10] presented a novel micropump in which the pumping mechanism is based upon magnetohydrodynamic (MHD) principles and showed experimentally that low-magnitude magnetic fields can be used to significantly increase the average flow rates in micropumps. After that, Ho [11] studied the flow characteristics of an electromagnetohydrodynamic (EMHD) micropump through a rectangular duct. Moghaddam [12] investigated the effect of a high Hartmann number on the flowing problem for AC- and DC-powered MHD micropumps with a circular channel. Additionally, a DC MHD micropump that can operate at high DC current densities was demonstrated by Homsy et al. [13]. Gao and Jian [14] studied the MHD flow of Jeffrey fluid in a circular microchannel and investigated the effect of the Hartmann number, dimensionless relaxation time, and retardation time on the velocity and volumetric flow rate.

Most previous research has focused on smooth channels. The walls of actual channels are rough as a result of manufacturing processes or the adsorption of other species like macromolecules. Since the 1970s, many analytical and computational approaches for studying the effect of wall roughness on laminar flow have been developed.

Chen et al. [15] investigated the mixing characteristics of electrokinetically-driven flow in microchannels with different wavy surface configurations. Numerical simulations were performed to analyze the influence of the wave amplitude and the length of the wavy section on the mixing efficiency within the microchannel. The research indicated that surface roughness can be designed artificially to promote mixing. Nishimura et al. [16] dealt with mass-transfer enhancement and fluid dynamic behavior in a wavy-walled tube for pulsatile flow. These results indicate that the transport enhancement mechanism for the wavy-walled tube is quite different from the resonant transport enhancement observed in the two-dimensional (2-D) wavy-walled channel, and a wavy-walled tube can enhance mass transfer through special enhancement mechanisms. Ligrani et al. [17] discussed the effects of near-wall slip induced by surface roughness on the pressure rise and flow rate of a single rotating-disk viscous micropump operating with Newtonian water. The Stokes flow problem of viscous fluid in corrugated pipes was considered by Phan-Thien [18]. Chu [19] investigated the effect of surface roughness on slip flow in an annulus with corrugated walls. Grant Mills et al. [20] studied the influence of a channel with sinusoidal walls on the unsteady laminar flow of a Newtonian fluid and established the optimum wall amplitude and period leading to an unsteady flow at the minimum pressure gradient. Numerous studies on electromagnetic flow in rough channels have been conducted. Buren et al. [21] studied the effect of the channel with parallel surface roughness on the EMHD flow. They investigated the impact of roughness on the flow rate. Furthermore, the impact of a channel with transverse surface roughness on EMHD flow was investigated by Buren and Jian [22].

Typically, a sinusoidal function was used to represent the wall roughness. However, during real manufacturing, the wall roughness is frequently random. Therefore, it is necessary to expand the investigations to include random roughness in addition to sinusoidal roughness. Phan-Thien [23] was the first to apply the theory of stationary random process to the Stokes flow of incompressible Newtonian fluids between parallel plates with transverse stationary random surface roughness. The results were in strong accord with those of Wang [24] when the wall roughness reduces to the sinusoidal one. Following that, Phan-Thien [25] extended the previous results to channels and pipes with parallel stationary random surface roughness. Faltas et al. [26] investigated the influence of stationary random surfaces on the modified micropolar Brinkman model and particularly evaluated the influence of the corrugations on the pressure gradient and the flow rate. Very recently, by utilizing the perturbation method based upon stationary random function theory, Ma et al. [27] studied the effect of small random transverse wall roughness in a parallel plate microchannel on Electromagnetohydrodynamic (EMHD) flow.

However, the problem of the effect of random roughness on the electromagnetic flow in a micropipe has not been investigated. By utilizing the characteristics of stationary random processes, the small parameter expansion approach is primarily employed in this research to investigate the impact of random roughness on the electromagnetic flow in micropipes. The analytical solution until second order velocity is achieved in Section 2. Two specific examples, i.e., sinusoidal corrugation and triangular corrugation, are taken into consideration in Section 3. The effect of dimensionless parameters on the roughness function in the above two special examples is investigated in Section 4. Section 5 concludes the study.

## 2. Mathematical Formulation

**Figure 1:**
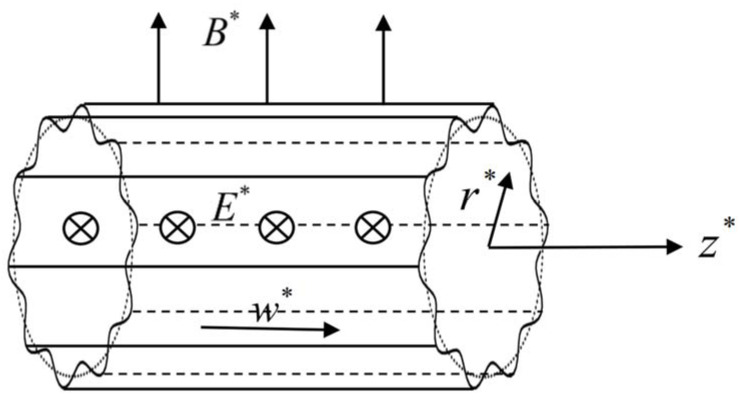
Sketch of the EMHD flow inside a micropipe with stationary random surface roughness.

The EMHD flow inside a micropipe with stationary random surface roughness is shown in Figure 1. The cylindrical coordinate system (r*, *θ*, z*) is established, and the mean radius is *R*. The pipe is exposed to orthogonal magnetic and electric fields. The magnetic induction intensity B* of the constant magnetic field is oriented vertically upward with the magnitude *B*_0_, and the direction of the electric field strength E* with the magnitude *E*_0_ is from the outside to the inside. The combined effect of the electric and magnetic fields produces the Lorentz force J* × B* in the z-axis. The current density is J* = *σ*(E* + u* × B*), where *σ* stands for conductivity, and the velocity in cylindrical coordinate system is given as u*(u*,v*,w*). It is assumed that the fluid flow is along the z-direction; i.e., u* = 0, v* = 0, and w*(r*,*θ*) ≠ 0, and the velocity w* in the z-direction is the function of r* and *θ* for steady flow. For a small magnetic Reynolds number, the momentum equation and induced magnetic field equation can be decoupled, and the Navier–Stokes equation of MHD flow in the micropipe can be expressed as

(1) $$\frac{\partial^{2}w^{*}}{\partial r^{*2}}+\frac{1}{r^{*}}\frac{\partial w^{*}}{\partial r^{*}}+\frac{1}{r^{*2}}\frac{\partial^{2}w^{*}}{\partial \theta^{2}}-\frac{\sigma B_{0}{}^{2}}{\mu }w^{*}=\frac{P^{*}-\sigma E_{0}B_{0}}{\mu },$$

where *μ* is the dynamic viscosity, $P^{*}=\partial {\bar{p}}^{*}/\partial x^{*}$ is the pressure gradient, ${\bar{p}}^{*}=p^{*}-\mu_{m}{\left|B_{0}\right|}^{2}/8\pi$ [28] is the modified pressure by magnetic pressure, and *μ_m_* is the magnetic permeability. The rough wall of the micropipe can be written as $R\left[1+\varepsilon n\left(\theta \right)\right]$, where *n*(*θ*) is a random process, and ε≪1 is a perturbation parameter. The no-slip boundary condition at $r^{*}=R\left[1+\varepsilon n\left(\theta \right)\right]$ can be expressed as

(2) $$w^{*}\left[R\left(1+\varepsilon n\left(\theta \right)\right),\theta \right]=0.$$

Assuming that *n*(*θ*) is a stationary random process and its mean is zero:

(3) $$\langle n\left(\theta \right)\rangle =0,$$

and the spectral representation of *n*(*θ*) by Fourier–Stieltjes integrals is:

(4) $$n\left(\theta \right)=\int_{-\infty }^{+\infty }e^{i\omega \theta }dh\left(\omega \right),$$

where *ω* is the frequency domain parameter, and *h*(*ω*) is the spectral process of the *n*(*θ*). The spectral process increment *dh*(*ω*) has the following properties:

(5) $$\langle dh\left(\omega \right)\rangle =0,$$

(6) $$\langle dh\left(\omega \right)\bar{dh\left(\omega^{\prime }\right)}\rangle =\left\{\begin{array}{cc} 0 & \;if\;\omega \neq \omega^{\prime }\\ dF\left(\omega \right) & \;if\;\omega \neq \omega^{\prime } \end{array}\right.,$$

where the ˉ represents the complex conjugate, and *F*(*ω*) is a function defined by the correlation function of *n*(*θ*).

(7) $$C\left(\theta -\theta^{’}\right)=\langle n\left(\theta \right)\bar{n\left(\theta^{\prime }\right)}\rangle =\int_{-\infty }^{+\infty }e^{i\omega \left(\theta -\theta^{\prime }\right)}dF\left(\omega \right),$$

The correlation function of *n*(*θ*) can be expressed as a Fourier integral:

(8) $$C\left(\tau \right)=\int_{-\infty }^{+\infty }e^{i\omega \tau }f\left(\omega \right)d\omega .$$

*f*(*ω*) is the spectral density of *n*(*θ*), expressed as

(9) $$f\left(\omega \right)=\frac{1}{2\pi }\int_{-\infty }^{+\infty }e^{-i\omega \tau }C\left(\tau \right)d\tau .$$

Then, the relation (6) becomes

(10) $$\langle dh\left(\omega \right)\bar{dh\left(\omega^{\prime }\right)}\rangle =\left\{\begin{array}{cc} 0 & \;if\;w\neq w^{\prime }\\ f\left(\omega \right)d\omega & \;if\;w=w^{\prime } \end{array}\right.\;$$

Assuming that the scale of velocity is known to be *U*, utilizing *R* and *μU*/2*R*^2^ as the length and pressure scales, respectively, the nondimensional governing equations of (1) and (2) become

(11) $$\frac{\partial^{2}w}{\partial r^{2}}+\frac{1}{r}\frac{\partial w}{\partial r}+\frac{1}{r^{2}}\frac{\partial^{2}w}{\partial \theta^{2}}-Ha^{2}w=P-HaS,$$

(12) $$w\left[1+\varepsilon n\left(\theta \right)\right]=0,$$

where $S=\frac{RE_{0}}{U}\sqrt{\frac{\sigma }{\mu }}$, $Ha=RB_{0}\sqrt{\frac{\sigma }{\mu }}$, and $P=\frac{R^{2}P^{*}}{\mu U}$ are the dimensionless electric parameter, the Hartmann number, and the dimensionless pressure gradient, respectively. Using the small parameter expansion method, the solution can be assumed as the form

(13) $$w\left(r,\theta \right)=w_{0}\left(r,\theta \right)+\varepsilon w_{1}\left(r,\theta \right)+\varepsilon^{2}w_{2}\left(r,\theta \right)+O\left(\varepsilon^{3}\right),$$

By substituting (13) into (11), the governing equations for powers of *ε* are as follows:

(14) $$\varepsilon^{0}\text{:}\;\frac{\partial^{2}w_{0}}{\partial r^{2}}+\frac{1}{r}\frac{\partial w_{0}}{\partial r}-Ha^{2}w_{0}=P-HaS,$$

(15) $$\varepsilon^{1}\text{:}\;\frac{\partial^{2}w_{1}}{\partial r^{2}}+\frac{1}{r}\frac{\partial w_{1}}{\partial r}+\frac{1}{r^{2}}\frac{\partial^{2}w_{1}}{\partial \theta^{2}}-Ha^{2}w_{1}=0,$$

(16) $$\varepsilon^{2}\text{:}\;\frac{\partial^{2}w_{2}}{\partial r^{2}}+\frac{1}{r}\frac{\partial w_{2}}{\partial r}+\frac{1}{r^{2}}\frac{\partial^{2}w_{2}}{\partial \theta^{2}}-Ha^{2}w_{2}=0.$$

By substituting (13) into the boundary conditions (12) and employing the Taylor expansion for the average position of the pipe walls (*r* = 1), we obtain the boundary condition as

(17) $$w_{0}\left(1\right)+\varepsilon \{w_{1}\left(1,\theta \right)+n\frac{\partial w_{0}\left(1\right)}{\partial r}\}+\varepsilon^{2}\{w_{2}\left(1,\theta \right)+n\left(\theta \right)\frac{\partial w_{1}\left(1,\theta \right)}{\partial r}+\frac{1}{2}n^{2}\left(\theta \right)\frac{\partial^{2}w_{0}\left(1\right)}{\partial r^{2}}\}+O\left(\varepsilon^{3}\right)=0.$$

On collecting terms of equal powers of *ε*, we obtain the boundary conditions for Equations (14)–(16), respectively:

(18) $$w_{0}\left(1\right)=0,$$

(19) $$w_{1}\left(1,\theta \right)=-n\left(\theta \right)\frac{\partial w_{0}\left(1\right)}{\partial r},$$

(20) $$w_{2}\left(1,\theta \right)=-n\left(\theta \right)\frac{\partial w_{1}\left(1,\theta \right)}{\partial r}-\frac{1}{2}n^{2}\left(\theta \right)\frac{\partial^{2}w_{0}\left(1\right)}{\partial r^{2}}.$$

From Equations (14) and (18), the leading-order solution can be solved as

(21) $$w_{0}(r)=\frac{HaS-P}{Ha^{2}}\left(1-\frac{I_{0}\left(Ha\cdot r\right)}{I_{0}\left(Ha\right)}\right),$$

where *I_k_* represents the first kind of modified Bessel function of the order *k*.

Substituting the leading-order solution into condition (19), the second-order boundary condition can be expressed as

(22) $$w_{1}\left(1,\theta \right)=n\left(\theta \right)\frac{\left(HaS-P\right)I_{1}\left(Ha\right)}{Ha\cdot I_{0}\left(Ha\right)}=\frac{\left(HaS-P\right)I_{1}\left(Ha\right)}{Ha\cdot I_{0}\left(Ha\right)}\int_{-\infty }^{+\infty }e^{i\omega \theta }dh\left(\omega \right)$$

The first order solution *w*_1_(*r*,*θ*) is considered as a spectral representation, which is suggested by the condition (22)

(23) $$w_{1}\left(r,\theta \right)=\frac{\left(HaS-P\right)I_{1}\left(Ha\right)}{Ha\cdot I_{0}(Ha)}\int_{-\infty }^{+\infty }\varphi_{1}\left(r\right)e^{i\omega \theta }dh\left(\omega \right).$$

The ordinary differential equation with respect to *φ*_1_ is obtained by substituting the solution (23) into the first-order Equation (15):

(24) $$\frac{d^{2}\varphi_{1}}{dr^{2}}+\frac{1}{r}\frac{d\varphi_{1}}{dr}-\left(\frac{\omega^{2}}{r^{2}}+Ha^{2}\right)\varphi_{1}=0.$$

The general solution of Equation (24) is

(25) $$\varphi_{1}\left(r,\omega \right)=AI_{\omega }\left(Ha\cdot r\right)+BK_{\omega }\left(Ha\cdot r\right),$$

where *A* and *B* are arbitrary constants. The boundary condition of Equation (24) is

(26) $$\varphi_{1}\left(1,\omega \right)=1.$$

Since *φ*_1_(0,*ω*) has a finite value, and *K_ω_*(0) trends to infinite, *B* is equal to 0. From condition (26) and general solution (25), Equation (24) can be solved as

(27) $$\varphi_{1}\left(r,\omega \right)=\frac{I_{\omega }(Ha\cdot r)}{I_{\omega }(Ha)}.$$

Substituting expression (27) into (23), the analytic expression of *w*_1_(*r*,*θ*) becomes

(28) $$w_{1}\left(r,\theta \right)=\frac{\left(HaS-P\right)I_{1}\left(Ha\right)}{Ha\cdot I_{0}(Ha)}\int_{-\infty }^{+\infty }\frac{I_{\omega }(Ha\cdot r)}{I_{\omega }(Ha)}e^{i\omega \theta }dh\left(\omega \right).$$

Taking the leading-order and first-order solutions into condition (20), the second-order boundary condition is written as

(29) $$w_{2}\left(1,\theta \right)=\frac{HaS-P}{4I_{0}\left(Ha\right)}\int_{-\infty }^{+\infty }\int_{-\infty }^{+\infty }D\left(\omega \right)e^{i\left(\omega -\omega^{\prime }\right)\theta }dh\left(\omega \right)\bar{dh\left(\omega^{\prime }\right)},$$

where *D*(*ω*) is

(30) $$D\left(\omega \right)=-2I_{1}\left(Ha\right)\frac{I_{\omega -1}(Ha)+I_{\omega +1}(Ha)}{I_{\omega }(Ha)}+I_{2}(Ha)+I_{0}(Ha).$$

The second order solution *w*_2_(*r*,*θ*) is considered as a spectral representation, which is suggested by the condition (29):

(31) $$w_{2}\left(r,\theta \right)=\frac{HaS-P}{4I_{0}\left(Ha\right)}\int_{-\infty }^{+\infty }\int_{-\infty }^{+\infty }\varphi_{2}(r,\omega )e^{i\left(\omega -\omega^{\prime }\right)\theta }dh\left(\omega \right)\bar{dh\left(\omega^{\prime }\right)}.$$

The ordinary differential equation satisfied by *φ*_2_(*r*) can be obtained by substituting Equation (31) into Equation (16):

(32) $$\frac{d^{2}\varphi_{2}}{dr^{2}}+\frac{1}{r}\frac{d\varphi_{2}}{dr}-\left[\frac{{\left(\omega -\omega^{\prime }\right)}^{2}}{r^{2}}+Ha^{2}\right]\varphi_{2}=0.$$

The general solution of Equation (32) is

(33) $$\varphi_{2}\left(r,\omega \right)=AI_{\omega -\omega^{\prime }}\left(Ha\cdot r\right)+BK_{\omega -\omega^{\prime }}\left(Ha\cdot r\right),$$

where *A* and *B* are arbitrary constants. The boundary condition of Equation (30) is

(34) $$\varphi_{2}\left(1,\omega \right)=D\left(\omega \right).$$

Similarly, since *φ*_2_(0,*ω*) has a finite value, *B* is equal to 0. From condition (34) and general solution (33), the solution of Equation (32) can be written as

(35) $$\varphi_{2}\left(r,\omega \right)=\frac{D\left(\omega \right)}{I_{\omega -\omega^{\prime }}\left(Ha\right)}I_{\omega -\omega^{\prime }}\left(Ha\cdot r\right).$$

Substituting expression (35) into (31), the analytic expression of *w*_2_(*r*,*θ*) becomes

(36) $$w_{2}\left(r,\theta \right)=\frac{HaS-P}{4I_{0}\left(Ha\right)}\int_{-\infty }^{+\infty }\int_{-\infty }^{+\infty }\frac{D\left(\omega \right)}{I_{\omega -\omega^{\prime }}\left(Ha\right)}I_{\omega -\omega^{\prime }}\left(Ha\cdot r\right)e^{i\left(\omega -\omega^{\prime }\right)\theta }dh\left(\omega \right)\bar{dh\left(\omega^{\prime }\right)}.$$

The rate of flow per unit length in the micropipe is obtained as

(37) $$Q=\int_{0}^{1+\varepsilon n\left(\theta \right)}\left(w_{0}\left(r\right)+\varepsilon w_{1}\left(r,\theta \right)+\varepsilon^{2}w_{2}\left(r,\theta \right)\right)rdr.$$

The formula (37) can be expanded in the Taylor series about the mean wall positions *r* = 1:

(38) $$\begin{array}{c} Q=\int_{0}^{1}w_{0}\left(r\right)rdr+\varepsilon \left(\int_{0}^{1}w_{1}\left(r,\theta \right)rdr+w_{0}\left(1\right)n\left(\theta \right)\right)+\\ \varepsilon^{2}\left(\int_{0}^{1}w_{2}\left(r,\theta \right)rdr+w_{1}\left(1,\theta \right)n\left(\theta \right)+\frac{dw_{0}\left(1\right)}{dr}\frac{n{\left(\theta \right)}^{2}}{2}\right). \end{array}$$

Taking the average of formula (38), we obtain the expression as

(39) $$\langle Q\rangle =\int_{0}^{1}w_{0}\left(r\right)rdr+\varepsilon^{2}\left(\langle \int_{0}^{1}w_{2}\left(r,\theta \right)rdr\rangle +\langle w_{1}\left(1,\theta \right)n\left(\theta \right)\rangle +\langle \frac{dw_{0}\left(1\right)}{dr}\frac{n{\left(\theta \right)}^{2}}{2}\rangle \right).$$

The portions of the second-order term of *ε* taking the mean value are shown, respectively,

(40) $$\begin{array}{cc} \langle \int_{0}^{1}w_{2}\left(r,\theta \right)rdr\rangle & =\int_{-\infty }^{+\infty }\int_{-\infty }^{+\infty }\frac{\left(HaS-P\right)D\left(\omega \right)}{4I_{0}{}^{2}\left(Ha\right)}\int_{0}^{1}I_{\omega -\omega^{\prime }}\left(Ha\cdot r\right)\cdot rdr\cdot e^{i\left(\omega -\omega^{\prime }\right)\theta }\langle dh\left(\omega \right)\bar{dh\left(\omega^{\prime }\right)}\rangle \\ & =\int_{-\infty }^{+\infty }\frac{\left(HaS-P\right)I_{1}\left(Ha\right)D\left(\omega \right)}{4Ha\cdot I_{0}{}^{2}\left(Ha\right)}f\left(\omega \right)d\omega \end{array},$$

(41) $$\begin{array}{cc} \langle w_{1}\left(1,\theta \right)\cdot n\left(\theta \right)\rangle & =\int_{-\infty }^{+\infty }\int_{-\infty }^{+\infty }\frac{\left(HaS-P\right)I_{1}\left(Ha\right)}{Ha\cdot I_{0}\left(Ha\right)}e^{i\left(\omega -\omega^{\prime }\right)\theta }\langle dh\left(\omega \right)\bar{dh\left(\omega^{\prime }\right)}\rangle \\ & =\int_{-\infty }^{+\infty }\frac{\left(HaS-P\right)I_{1}\left(Ha\right)}{Ha\cdot I_{0}\left(Ha\right)}f\left(\omega \right)d\omega , \end{array}$$

(42) $$\begin{array}{cc} \langle \frac{dw_{0}\left(1\right)}{dr}\frac{n{\left(\theta \right)}^{2}}{2}\rangle & =\int_{-\infty }^{+\infty }\int_{-\infty }^{+\infty }-\frac{\left(HaS-P\right)I_{1}\left(Ha\right)}{2Ha\cdot I_{0}\left(Ha\right)}e^{i\left(\omega -\omega^{\prime }\right)\theta }\langle dh\left(\omega \right)\bar{dh\left(\omega^{\prime }\right)}\rangle \\ & =\int_{-\infty }^{+\infty }-\frac{\left(HaS-P\right)I_{1}\left(Ha\right)}{2Ha\cdot I_{0}\left(Ha\right)}f\left(\omega \right)d\omega . \end{array}$$

The mean rate of flow *Q*_0_ in a smooth micropipe is described as

(43) $$Q_{0}=\int_{0}^{1}w_{0}\left(r\right)rdr=\frac{HaS-P}{2Ha^{2}}-\frac{(HaS-P)I_{1}\left(Ha\right)}{Ha^{3}I_{0}\left(Ha\right)}.$$

Formula (39) can be arranged in the form of

(44) $$\frac{\langle Q\rangle }{Q_{0}}=1-\varepsilon^{2}\zeta \left(Ha\right),$$

where $\zeta \left(Ha\right)=\int_{-\infty }^{+\infty }g\left(Ha,\omega \right)f\left(\omega \right)d\omega$ is defined as a deviation in the flow rate ratio with roughness to the case having no roughness in a smooth micropipe and

(45) $$g\left(Ha,\omega \right)=\frac{Ha^{2}I_{1}\left(Ha\right)\left[2I_{1}\left(Ha\right)\cdot \left(I_{\omega -1}\left(Ha\right)+I_{\omega +1}\left(Ha\right)\right)-I_{\omega }\left(Ha\right)\cdot \left(I_{2}\left(Ha\right)+I_{0}\left(Ha\right)\right)\right]}{2I_{0}\left(Ha\right)\cdot I_{\omega }\left(Ha\right)\cdot \left(Ha\cdot I_{0}\left(Ha\right)-2I_{1}\left(Ha\right)\right)}.$$

Assuming *n*(*θ*) holds Dirichlet’s condition, it can be expressed as a Fourier sinusoidal series:

(46) $$n\left(\theta \right)=\sum_{k=1}^{\infty }b_{k}\sin\left(k\lambda \theta \right),$$

where *b_k_* is the amplitude, and *λ* is the wave number. The correlation function and spectral density function of *n*(*θ*), respectively, are [22,23,24]

(47) $$C\left(\tau \right)=\langle n\left(\theta \right)n\left(\theta +\tau \right)\rangle =\frac{1}{2}\sum_{k=1}^{\infty }b_{k}^{2}\cos\left(k\lambda \tau \right),$$

(48) $$f\left(\omega \right)=\frac{1}{2\pi }\int_{-\infty }^{+\infty }e^{-i\omega \tau }C\left(\tau \right)d\tau =\frac{1}{4}\sum_{k=1}^{\infty }b_{k}^{2}\left(\delta \left(\omega -k\lambda \right)+\delta \left(\omega +k\lambda \right)\right),$$

where *δ*(·) is the generalized Dirac function.

## 3. Two Special Examples

### 3.1. Sinusoidal Corrugation

**Figure 2:**
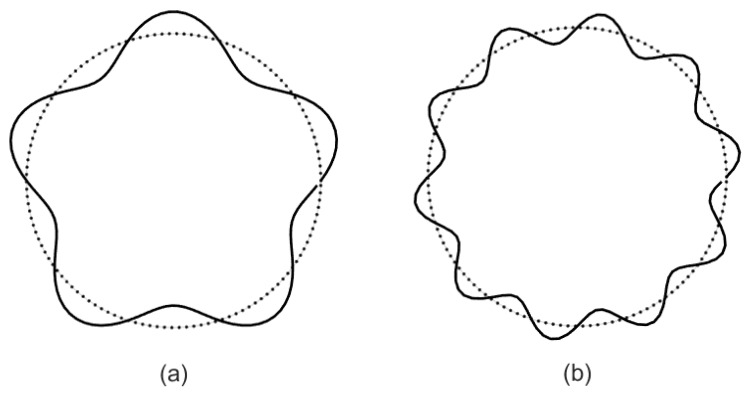
Cross section of a cylindrical pipe with sinusoidal roughness. (**a**) *ε* = 0.15, *λ* = 5, (**b**) *ε* = 0.1, *λ* = 10.

Using the results of the prior investigation, we consider two types of corrugations. The stationary random process *n*(*θ*) reduces to sin(*λθ*) when the micropipe has sinusoidal corrugation. According to (46), we can obtain that *b*_1_ = 1 and *b_k_* = 0, *k* ≥ 2 for *n*(*θ*) = sin(*λθ*) (Figure 2). Formula (44) in this situation can be written as

(49) $$\frac{\langle Q\rangle }{Q_{0}}=1-\varepsilon^{2}\zeta_{1}(Ha,\lambda ),$$

(50) $$\zeta_{1}=\frac{1}{2}g\left(Ha,\lambda \right).$$

### 3.2. Triangular Corrugation

**Figure 3:**
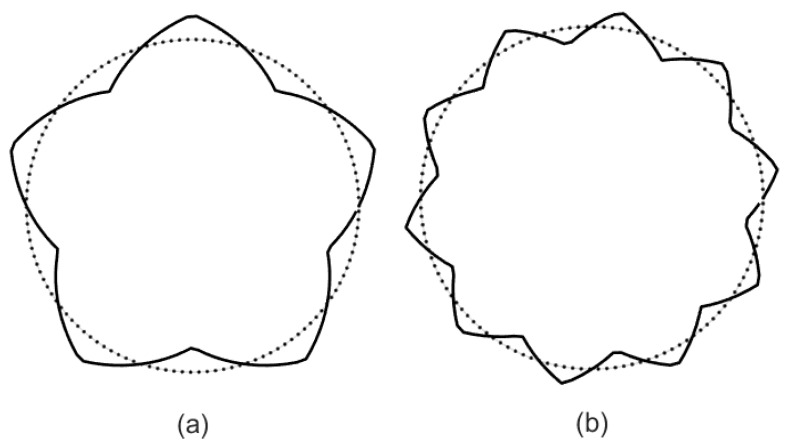
Cross section of a cylindrical pipe with triangular roughness. (**a**) *ε* = 0.15, *λ* = 5, (**b**) *ε* = 0.1, *λ* = 10.

When the micropipe has triangular corrugation (Figure 3), the stationary random process *n*(*θ*) reduces to

(51) $$n\left(\theta \right)=\left\{\begin{array}{c} \begin{array}{c} \frac{2}{\pi }\lambda \theta \\ 2-\frac{2}{\pi }\lambda \theta \end{array}\\ \frac{2}{\pi }\lambda \theta -4 \end{array}\right.\begin{array}{c} \begin{array}{c} 0<\theta <\frac{\pi }{2\lambda }\\ \frac{\pi }{2\lambda }<\theta <\frac{3\pi }{2\lambda } \end{array}\\ \frac{3\pi }{2\lambda }<\theta <\frac{2\pi }{\lambda } \end{array}.$$

Expanding (51) into a sinusoidal series, the radius of the micropipe with triangular corrugation is obtained:

(52) $$r=1+\varepsilon \sum_{n=1}^{\infty }b_{n}\sin\left(2n-1\right)\lambda \theta ,$$

where *b_n_* = 8(−1)*^n^*^− 1^/(*π*^2^(2*n* − 1)^2^). Formula (44) in this situation can be written as

(53) QQ0=1−ε2ζ2Ha,λ,

(54) $$\zeta_{2}=\frac{1}{2}\sum_{k=1}^{\infty }\frac{64g\left(Ha,\lambda \right)}{\pi^{4}{\left(2n-1\right)}^{4}}$$

## 4. Results and Discussion

Based on the reference [29], the range of dimensionless parameters is estimated as follows. The radius of the pipe is *R*~10–500 μm, viscosity *μ*~10^−3^–1.5 × 10^−3^ kg/(ms), electrical conductivity *σ*~2.2 × 10^−4^ S/m, and the strength of magnetic field *B*~0.01–1 T. The order of the Hartmann number *O*(*Ha*) ranges from 0 to 5, and the order of S ranges from 0 to 6 × 10^3^.

**Figure 4:**
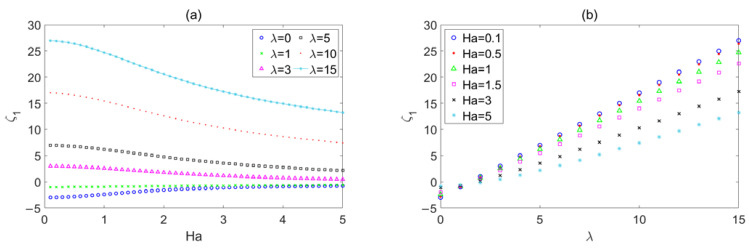
The variations in the sinusoidal corrugation function *ζ*_1_ with (**a**) *Ha* for different *λ*, (**b**) *λ* for different *Ha*.

The influence of *Ha* on *ζ*_1_ is shown in Figure 4a, when the wave number *λ* = 0, 1, 3, 5, 10, and 15. A larger wave number means a rougher wall. *ζ*_1_ represents the deviation between the flow rate of a rough wall with sinusoidal corrugation and the flow rate of a smooth wall. *ζ*_1_ is positive, and it approaches 0 with the increase in *Ha* when the wave number is 3, 5, 10, and 15, which indicates that roughness has an impeding effect on flow, and the effect is gradually weakened with the increase in *Ha*. This phenomenon also occurs in the electromagnetic flow between microparallel plates with transversely wavy surfaces [22] and electromagnetic flow in microchannels with random surface roughness [27]. In particular, when the wave number is 0 and 1, the deviation is negative, and it approaches 0 with the increase in *Ha*, which means that roughness has a promoting effect on the flow, and the effect also weakens with the increase in *Ha*.

The influence of *λ* on *ζ*_1_ is shown in Figure 4b when the Hartmann number *Ha* = 0.1, 0.5, 1, 1.5, 3, and 5. The deviation *ζ*_1_ progressively increases from zero with the increase in wave number when the wave number is greater than 2. Since the deviation *ζ*_1_ is positive, it is clear that roughness impedes the flow on micropipe, and this effect steadily reinforces as the wave number grows. This conclusion is consistent with the conclusion in reference [22,27]. The deviation *ζ*_1_ approaches to 0 from a negative value with an increase in *λ* from 0 to 2, which also means that the roughness has a promoting effect on the flow, and the effect weakens with an increase in the wave number. A similar phenomenon also appears in Ref. [26], perhaps because the method has certain errors in the case of a small wave number.

**Figure 5:**
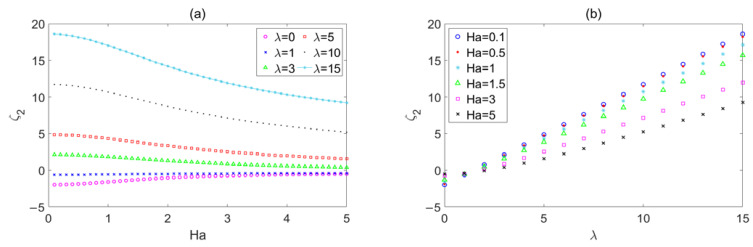
The variations in the triangular corrugation function *ζ*_2_ with (**a**) *Ha* for different *λ*, (**b**) *λ* for different *Ha*.

**Figure 6:**
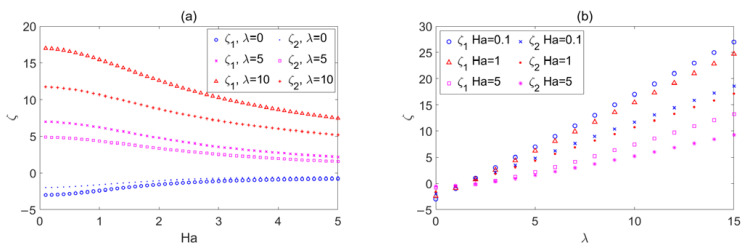
(**a**) depicts the change curves of *ζ*_1_ and *ζ*_2_ in relation to *Ha* when *λ* = 0, 5, and 10; (**b**) depicts the change curves of *ζ*_1_ and *ζ*_2_ in relation to *λ* when *Ha* = 0.1, 1, and 5.

The influence of *Ha* on *ζ*_2_ is shown in Figure 5a when the wave number *λ* = 0, 1, 3, 5, 10, and 15. *ζ*_2_ represents the deviation between the flow rate of a rough wall with triangular corrugation and the flow rate of a smooth wall. The influence of *λ* on *ζ*_2_ is shown in Figure 5b when *Ha* = 0.1, 0.5, 1, 1.5, 3, and 5. There are a few differences between Figure 4 and Figure 5. Figure 6 compares the two cases in detail.

The influence of *Ha* on deviation *ζ*_1_ and *ζ*_2_ with *Ha* when *λ* = 0, 5, and 10 is depicted in Figure 6a. Figure 6b depicts the influence of *λ* on deviation *ζ*_1_ and *ζ*_2_ when *Ha* = 0.1, 1, and 5. The graph indicates that *ζ*_1_ is greater than *ζ*_2_ for the same *Ha* and *λ*. This means that compared with triangular corrugation, sinusoidal corrugation has a larger resistant effect on flow. This conclusion is similar to the conclusion in Ref. [26].

**Figure 7:**
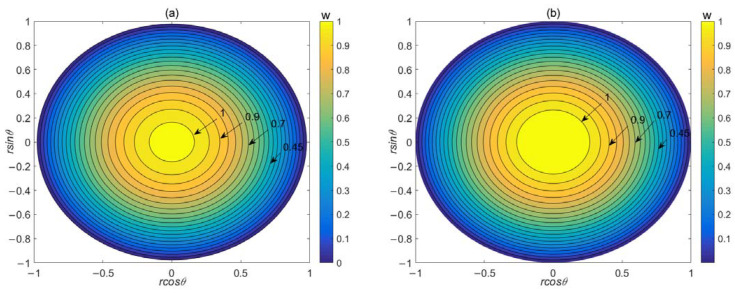
Mean velocity distribution in the micropipe with (**a**) sinusoidal corrugation and (**b**) triangular corrugation. *P* = −0.5, *S* = 8, *Ha* = 0.5, *λ* = 5, *ε* = 0.1.

Figure 7 depicts the mean velocity distribution in the micropipe with both sinusoidal corrugation and triangular corrugation when *P* = −0.5, *S* = 8, *Ha* = 0.5, *λ* = 5, and *ε* = 0.1. Taking the average of velocity is essentially equivalent to taking the average of the surface roughness. This method is essentially used to transform the flow problem within micropipes with roughness into the problem within smooth micropipes. Because different roughness types have different effects on the flow inside the micropipes, this leads to different velocity distributions. The velocity distribution we obtained is actually the corrected velocity distributions of different types of roughness within smooth micropipes. From Figure 7a,b, it can be observed that at the same dimensionless parameters, the velocity distribution within micropipes with triangular corrugation is generally larger than that within micropipes with sinusoidal corrugation. The reason for this is that sinusoidal corrugation has a stronger influence on the flow rate compared with triangular corrugation. Under the same corrugation parameters, sinusoidal corrugation has a larger resistant effect on flow, resulting in lower flow velocities. This more intuitively demonstrates our conclusion about the influence of these two types of corrugated surfaces on flow rate.

## 5. Conclusions

The effect of random roughness on the electromagnetic flow in a micropipe is investigated using random function theory. The random corrugation function *ζ*, which is a measure of the flow rate deviation from the situation with no pipeline roughness, is analytically expressed by expanding the velocity with small parameters and using some stationary random process features. Two particular micropipes with sinusoidal and triangular corrugations are explored in this work. By examining graphical fluctuations in the roughness function concerning the Hartmann number *Ha* and wave number *λ*, it is revealed that the effect of roughness on the flow rate decreases with an increase in *Ha* and increases with an increase in the wave number *λ*. The sinusoidal corrugation has a stronger influence on the flow rate under the same values of *Ha* and *λ* compared with triangular corrugation. The mean velocity distribution indicates that the overall velocity within the micropipe with sinusoidal corrugation is relatively low. This is primarily due to the larger impact of sinusoidal corrugation on the flow rate. Meanwhile, this implies that the influence in obstructing the flow is more pronounced. This method is essentially used to transform the flow problem within micropipes with roughness into the problem within smooth micropipes to study the influence of roughness on the flow rate, and the velocity distribution will be different according to the different roughness types. This method is not only effective for a given corrugation but also effective for random roughness.

## Data Availability

Data sharing not applicable.

## Abbreviations

The bold font represents vectors and the normal font represents scalars.

(*r,θ,x*)	cylindrical coordinate system
***B***	magnetic field
***B*_0_**	the magnitude of the magnetic field
***E***	electric field
***E*_0_**	the magnitude of the electric field
***J***	electrical current density
***u***	velocity vector
*σ*	specific conductance
*u*, *v*, *w*	components of velocity vector
***p***	pressure of the liquid
*R*	channel radius
*μ*	dynamic viscosity
*n*(*θ* )	stationary random functions
*ε*	amplitude of the corrugation
*dh*(*ω*)	interval random function
*F*(*ω*)	real non-decreasing bounded functions
*f*(*ω*)	spectral densities of *n*(*θ*)
*C*	correlation function
*I_n_*	modified Bessel function of the first kind
*K_n_*	modified Bessel function of the second kind
*Ha*	Hartmann number
*S*	dimensionless electric field strength
*Q*	flow rate per unit length
*Q*_0_	the mean rate of flow in smooth micropipe
*ζ*	corrugation function
*λ*	wave number
*δ*	the Dirac generalized function
*θ*	phase difference
